# Family networks to improve outcomes in children with intellectual and developmental disorders: a qualitative study

**DOI:** 10.1186/1752-4458-8-7

**Published:** 2014-02-01

**Authors:** Syed Usman Hamdani, Najia Atif, Mahjabeen Tariq, Fareed Aslam Minhas, Zafar Iqbal, Atif Rahman

**Affiliations:** 1 Human Development Research Foundation, Islamabad, Pakistan; 2 Institute of Psychiatry, Rawalpindi, Pakistan; 3 Institute of Psychology, Health & Society Child Mental Health Unit Alder Hey Children’s NHS Foundation Trust, University of Liverpool, Mulberry House, Eaton Road, Liverpool L12 2AP, UK

**Keywords:** Intellectual or developmental disorders, Treatment gap, Task sharing approach, Family volunteers, Peer supervision

## Abstract

**Background:**

There are at least 50 million children with an intellectual or developmental disorder in South Asia. The vast majority of these children have no access to any service and there are no resources to develop such services. We aimed to explore a model of care-delivery for such children, whereby volunteer family members of affected individuals could be organized and trained to form an active, empowered group within the community that, a) using a task-sharing approach, are trained by specialists to provide evidence-based interventions to their children; b) support each other, with the more experienced FaNs i.e. family networks, providing peer-supervision and training to new family members who join the group; and c) works to reduce the stigma associated with the condition.

**Methods:**

We used qualitative methods to explore carers’ perspectives about such a care-delivery model.

**Results:**

The key findings of this research are that there is a huge gap between the needs of the carers and available services. Carers would welcome a volunteer-led service, and some community members would have time to volunteer. Raising community awareness in a culturally sensitive manner prior to launching such a service and linking it to the community health workers programme would increase the likelihood of success. Gender-matching would be important. It would be possible to form family networks around the more motivated volunteers, with support from local non-governmental organizations. The carers were receptive to the use of technology to assist the work of the volunteers as well as for networking.

**Conclusions:**

We conclude that family volunteers delivering evidence-based packages of care after appropriate training is a feasible system that can help reduce the treatment gap for childhood intellectual and developmental disorders in under-served populations.

## Background

Improving children’s access to evidence-based mental health care by trained health providers in low- and middle-income countries is one of the top five challenges for global mental health [1]. However, lack of trained human resource and financing for mental health have been identified as major, and intractable, barriers to the delivery of such care [2]. With an estimated population size of 180 million and approximately 45% of Pakistan’s population between the ages of 0–18, the estimated rates of intellectual disability are 1.9% for serious and 6.5% for mild intellectual disability [3-5]. Extrapolating these figures to the whole of South Asia, there are at least 50 million children with an intellectual or developmental disability in the region. Most of these children live in rural areas with no access to any form of specialist public or private service. Our work in rural Pakistan has shown that children with these disorders are misdiagnosed and mismanaged, leading to high levels of parental stress as well as a drain on family resources [6]. In Pakistan, only 2.7% of the GDP is allocated to health, and less than 1% of the health budget is allocated to all of mental health [7], it is unlikely there will ever be sufficiently enough trained child mental health providers to meet the needs of the population.

Community-Based Rehabilitation (CBR), advocated by the WHO in the last 3 decades, has been the preferred approach for community management of these disorders [8]. The focus of intervention is shifted from city based institutions to the community, involving people with disabilities, family members and volunteers, supported by local health professionals as well as having a system of consultation/referral to more specialized services. Subsequently, it has moved from being primarily a ‘medical model’ to a more ‘social model’ in recognition that a considerable contribution to disability is made by the attitude of society. This has lead to the move to minimize stigmatization and to support inclusive education and integration of people with disabilities into society [9].

The role of families is central for implementation of CBR approaches, and has been emphasized in children with disability in low income settings [10,11]. However, lack of local expertise has been identified as an important barrier to dissemination of such programmes [6]. The last decade has seen progress in the development of evidence-based mental health care packages that can be delivered by less-specialised professionals after brief training but under specialist supervision [12]. Termed ‘task-shifting’, such strategies have been tried successfully with a number of mental conditions. Task-shifting is a key strategy to reduce the treatment gap for mental disorders and the WHO mental health Gap Action Programme (mhGAP) has developed an evidence-based intervention guide designed for primary care staff including community health workers [13]. Our previous work has shown that community health workers (CHWs) can be trained to deliver community-based interventions for children with intellectual disorders [14].

However, it is problematic to scale up such services. Primary care systems in most low income countries are often fragmented and weak. CHWs, on which such programmes rely, are a finite resource, and are increasingly called upon to take on multiple public health roles. The over-burdening of community health workers has been recognised, both in Pakistan [15] and globally [16]. Meeting the Grand Challenges to child mental health clearly requires solutions which look beyond health care systems.

In this context, we are currently conducting a study to explore if task-shifting for the management of intellectual and developmental disorders can be taken to its most proximal level, i.e., family members of children with such disorders.

We aimed to explore a model of care-delivery for such children, whereby volunteer family members of affected individuals could be organized and trained to form an active, empowered group within the community that, a) using a task-sharing approach, are trained by specialists to provide evidence-based interventions to their children; b) support each other, with the more experienced Family Networker(FaNs) (family members of children with such disorders) providing peer-supervision and training to new family members who join the group; and c) works to reduce the stigma associated with the condition.

In this paper we report the findings from the formative phase of the project. Our aim was to explore the views of carers of children with such disorders about the feasibility of the above approach, the facilitators and barriers to such a system and how such an arrangement could be organised in one rural area of Pakistan.

## Methods

The study design was qualitative. The main objectives and research questions are summarised in Table 1.

**Table 1:** Objectives and research questions

**Objectives**	**Research questions**
Explore existing care practices for children with intellectual and developmental disorders in the community.	1: Who is the main carer for the child with intellectual and developmental disorders?
	2: What are the main problems experienced by parents in caring for such children?
	3: What type of care is sought and received for the child with developmental disorder?
	4: What are the community’s perception and attitude about children with developmental disorders?
Explore carers’ views about family volunteers to assist with care	1. How would the idea of family volunteers be received by carers?
	2. Who can take on the role of a family volunteer?
	3. What are the barriers to take on the role of a family volunteer and how could those barriers be overcome?
	4. What are the facilitators to take on the role of a family volunteer and how could these be integrated into the proposed system?
Explore potential family volunteers’ views about their role	1. How can family volunteers work with other families?
	2. How can family networks be formed and sustained?
	3. How can family volunteers be trained and supervised?
	4. How can technology assist in such a role?

### Settings and participants

The study was carried out in the rural Union Council (UC) of Mandra in the Rawalpindi District of Pakistan. A Union Council is the smallest rural administrative sub-division, consisting of about 10–15 villages with a population of about 1500–3000 per village. Mandra is located in sub-District Gujar Khan, about 35 Km South-East of the city of Rawalpindi. The economy is largely agrarian, with some men working in the nearby city or in the armed forces. Socially, families are often demarcated by kinship or caste (biradaris) and generally, a village is predominantly populated by people of the same biradari. Primary health care is organized through a network of Basic Health Units (BHU), one for each Union Council. A BHU is staffed by a doctor LHV, midwife, vaccinator and 15–20 village-based community health workers called Lady Health Workers (LHWs). LHWs have completed secondary school, and are trained to provide mainly preventive maternal and child health care and education in the community. Each LHW is responsible for about 100 households in her village. The nearest specialist care for mental health is the outpatients department at the Institute of Psychiatry, Rawalpindi.

In a previous study from an adjoining UC, we estimated there would be about 75 families with a child with an intellectual or developmental disorder in Mandra [17]. Such families were identified with the help of the LHWs who had information on all the households under their care. Participants for interview (mothers, fathers, siblings, aunts and grandmothers) were identified through purposive sampling. A total of 30 participants were interviewed, including 15 mothers, 6 fathers, 3 grandmothers, 3 grandfathers and 3 aunts.

### Data collection

Topic guides based on the research questions (Table 1) were prepared for the in-depth interviews, and piloted before data collection. Some additions were made and recorded in the topic guide iteratively during the data collection process to ensure all research questions were sufficiently addressed. Interviews were conducted at home or at a research office in Mandra, based on the participants’ preference. Participants were interviewed after informed written consent. Only those participants who voluntarily agreed to take part and who signed the consent form were interviewed. The duration of the interviews was between 60–90 minutes. Almost all of the interviews were recorded, except one, where participant did not allow the recording. Notes were taken during the interview. The recordings of the interviews were then transcribed by the same team members who conducted the interview. Data was collected until the saturation point was achieved.

### Data analysis

Data collection and data analysis were carried out simultaneously. Thematic analysis was used to analyze data. The transcribed data for each interview was read and reread to gain familiarity with the raw data. During the process of familiarization, the emerging categories were highlighted. These emerging categories were compared and contrasted with each other to identify any patterns in the raw data. A thematic table was developed to organize the emerging categories. Emerging categories that fitted under the similar pattern or meanings were placed within the corresponding patterns and coded (sub themes). Each interview was analysed by two researchers and then reviewed by a supervisor. Any discrepancies between analyses were discussed between the analysis team. These discussions were used as means of refining sub-themes. The sub-themes were analysed along with the supporting data (quotes) to fit together in a meaningful way to develop and interpret themes.

Ethical approval for the study was obtained from the Institutional Review Board (IRB) of the Human Development Research Foundation. As previously stated, carers were interviewed after obtaining written valid informed consent.

## Results

The family members interviewed had children ranging in age from 3 to 18 years, and roughly equal number of boys and girls. The house-hold income of the families were average for this region (about US$200 per month) and all except two lived in extended families living in the same or adjoined house. The level of intellectual disability ranged from mild to severe, but all the children were completely dependent on the family to meet their needs.

### Existing care-practices

In the majority of cases, all of the care was borne by the family and there was no formal service of any description to support these children or their families. The primary care-giver was generally the mother, with some respite care provided by other family members such as the older sibling, grandparent or aunts and uncles.

‘I have looked after him since his birth, and no one else understands his needs. My older daughter can manage him for a while, but he won’t eat if I don’t feed him.’ (Mother of an 8-year old boy)

In the absence of any state-provided respite care, it was left to the extended family to assist the mother.

‘If she (the mother) has to visit her parents, or attend some social occasion, then I take care of her. But she (the mother) is restricted in what she can do, and I feel sorry for her sometimes’. (Grandmother of a 12-year old girl)

Majority of the families interviewed had to deal with a range of problems in the children, which could roughly be divided into 3 broad themes – physical, behavioural and social. Physical problems included muscular weakness, limited mobility, bed-wetting and incontinence, and poorly controlled epilepsy. Problems with communicating and comprehending were common. Skin infections, dental problems and recurrent worm-infestations were frequently reported. Some parents felt the local doctors either lacked knowledge or were disinterested in helping such children.

‘The doctor told me that in such children, medicines don’t work, and he told me its best to pray to Allah to cure him’. (Father of a 13 year old boy with epilepsy)

Behavioural and social problems caused the most stress to the parents, particularly challenging behaviours that were socially embarrassing and contributed to stigma towards these children and their families. Several such behaviours were reported such as hitting others, screaming, crying, shouting, throwing and breaking things, and using abusive language towards others. Some behaviours were self-destructive, such as hitting and biting oneself.

‘When he gets angry he bites himself, bangs his head on the wall and if somebody goes near him he hits them too. No one comes near him’. (Mother of an 8-year old boy)

None of the parents were receiving any ongoing help or advice from an external agency to deal with these problems. They relied on advice from their elders, and other family members and sometimes from sympathetic community members. However, all of the advice was based on traditional folk wisdom. Faith figured prominently in the coping strategies employed. Faith healers were also regularly consulted who provided talisman and holy water.

‘Whenever she gets sick, I take her to the spiritual healer who gives a taweez (talisman). She gets better after wearing it’. (Mother of a 6 year old girl)

Almost all of the families had consulted a doctor on at least one occasion and many quite regularly, and for longer periods of time. None of the families interviewed reported any benefits from their interventions - which consisted mostly of psychopharmacology, multivitamins and physiotherapy.

‘For one year we visited the doctor, he used to give medicine but it did not work’. (Mother of an 11 year old boy)

‘They (doctors) say your child’s brain is weak but they do not guide us what to do’. (Grandmother of a 10 year old boy)

Most of the carers had no knowledge or understanding of the causes of the disorder and often attributed it to supernatural or religious phenomenon, such as the child being possessed by an evil spirit, the result of an evil eye, or a mother’s carelessness in pregnancy. Only a few had medically grounded beliefs such as the brain not being fully developed, consanguineous marriages over many generations, and brain injury or illness.

‘When he was born, his face was freckled and covered with dark circles. We thought that he is possessed by some evil spirit’. (Mother of a 6 year old girl)

‘People say that his parents must have committed sins so that is why their child is like this’. (Grandmother of a 5 year old boy)

The attitudes of the community towards such children were mixed. Many carers felt people were unsympathetic, intolerant and less understanding of such children’ needs. Many used derogatory terms such as pagal (mad) or Jhalla (idiot) for such children. However, others showed a positive attitude towards such children and were kind to the family.

‘Some people in the neighbourhood hate him. They never let their children play with my son. Even if he tries to play with them their parents stop them. Others are kind, and tell me these children are blessings from God’ (Father of a 12 year old boy)

### Views about family volunteers

There was a strong desire from the participants to learn more about how to deal with their children’s problems. There was great enthusiasm for a service that could provide such advice or training at their doorsteps. Quotes similar to the one below were echoed by many carers.

‘A bus journey to the city takes half a day. My son is sick from the journey and everyone stares at him. He is scared. The doctor tells us things which are hard to follow. We have wasted so much money and nothing gets better, so having someone to give us advice in our own village would be a blessing’. (Father of a 10 year old boy)

There was willingness to receive ‘training’ from a local person. Most of the participants were willing to be trained themselves, while some mothers indicted secondary carers (older sibling, aunt or grandparent) who would be more appropriate, primarily due to competing demands on the mother’s time.

‘His sisters and aunt usually teach him how to do things. They can be trained’. (Mother of a 12 year old boy)

The response from the carers about volunteering their own time to help other parents was less enthusiastic. The reasons cited were mostly practical. Some carers did not have the time due to their carer responsibilities in addition to domestic and other child-care responsibilities. The carers were mostly mothers, and many said that even if they had the time, it would be hard to get permission from the family to work outside the home or go out unaccompanied.

‘I have livestock and have to work in the field, and I have other domestic responsibilities too’.I don’t have time to help others’. (Mother of a 10 year old boy)

‘It will be difficult because I live in a joint family system. I cannot go outside without getting permission’. (Mother of a 6 year old girl)

There was an expectation that it was the government’s responsibility to provide such a service, and that people would be reluctant to work for nothing.

‘It should be the Lady Health Worker’s responsibility to get this training and then do this work for us. Can’t they be trained? No one will work for free’. (Father of a 6 year old girl)

However, some carers expressed their willingness to volunteer, or were able to identify a family member who they thought would be willing and able to volunteer their time for this work.

‘I think this would be similar to what the Lady health Worker does, except that it would be for special children like mine. I would like to do this, and if I were introduced to others by our Lady Health Worker, I think my husband would allow me. I could even take my daughter with me’. (Mother of a 10 year old girl)

‘His grandfather is retired. He has time, and he also likes to help others. I am sure he will be very interested’. (Mother of a 12 year old boy)

‘My niece wants to become a Health Worker. There are no jobs but I am sure she would love this experience. She is always helping me with my daughter’. (Mother of a 5 year old girl)

A number of potential community-level barriers towards such volunteer-work were described. Some of this was related to the social structures of villages in rural Punjab. The system of kinship or caste (‘biradari’) is prevalent in many rural areas and the status of a person is determined by his or her biradari. While many people no longer adhere to this system, some carers felt it would be difficult for a person from a lower biradari to be seen to ‘teach’ a person from a higher biradari. Some carers also thought people would be suspicious of persons if they did not know them.

‘If the volunteer was from a neechzaat (lower biradari), it would be hard for him to go into the house of a Chaudhri or Raja (higher biradari) and talk to the womenfolk’. (Grandfather of a 10 year old boy)

‘Here people are of a very suspicious nature. They will ask what is in it for this person. They always cook up stories out of nothing’. (Father of an 8 year old girl)

When asked how these barriers could be overcome, a number of useful themes emerged. Carers felt that the Lady Health Workers were now acceptable and well-regarded by most in the community. If the volunteers were seen to be working in partnership with the LHW programme, this would facilitate their work. The need to work on community awareness before starting the programme through meetings with the village elders was emphasised. There was also strong support for an activity that was perceived by all the participants to be altruistic and in accordance with their religious beliefs.

‘Allah blesses those who help others, so somebody who volunteers like this must be a good person. I am sure everyone will support them. We can ask the imam of the mosque to tell people to support this programme’. (Aunt of a 12 year old girl)

‘It will not look strange if the volunteers are introduced to the household by the Lady Health Workers as their assistants’. (Mother of a10 year old girl)

Another theme to emerge was related to the characteristics of a “good family volunteer”. The majority of participants felt that it was important for a volunteer to be good natured and kind, a good listener, and of good reputation in the community.

Gender matching was also felt to be very important – women volunteers to work with women and vice versa. Most carers felt the volunteer needed to be literate to be able to train or help others.

### Views about family networks

Most carers were comfortable with a volunteer contacting them through their mobile phone or visiting them at home. They were also comfortable contacting the volunteers through their own phone. However, some carers voiced reservations about going into somebody’s home, or conversely allowing someone into their homes, as it would contravene social convention. Many felt that a neutral venue, such as the local health centre, would be a good place to hold training events or a support group. Even for telephone calls, the majority view was that it would be important to match the gender of the caller and recipient.

‘People in my village are not educated – they will raise eyebrows if someone visits me at home regularly or if I go to someone’s place who is not related to me. However, no one will be bothered if I go to the health centre’. (Mother of a 6 year old girl)

‘If she is a woman I have no problem talking to her on phone and if it is a man my husband will speak to him’. (Mother of a 12 year old boy)

Most carers liked the idea of forming a group within their own village that could support each other and be able to call upon for advice and information. There was consensus such a group could also cooperate to improve opportunities for education and participation for their children. However, they felt that some organisation would have to assist them as they did not have the skills to organise themselves.

‘We can appoint a leader who can speak on our behalf. It is a good idea’. (Mother of 6 year old girl)

‘We don’t have the time to organise everyone to work together. We will need outside help’. (Father of a 6 year old girl)

Carers were interested to know how somebody just like them with no qualifications could give them useful advice. However, the majority agreed that they understood the problems of their own children more than any expert, and therefore could be trained with relative ease to help manage these problems, compared to a parent who had not had such an experience. They could then advise other carers how to manage these specific problems. All wanted the training to be simple and practical rather than just a lecture. Carers who were not literate felt they could benefit from DVDs (played on TV recorders), or pictorial and practical demonstrations, while those who could read said that training booklets would be helpful. People were very open to the idea of using available technology such as mobile phones and computers to assist such training. Continued supervision from experts was felt to be essential.

‘If someone taught me how to get my child to dress and use the toilet, I can teach others what I know’. (Mother of a 6 year old boy)

‘A CD would be really good because I can watch it with all my family and they too will learn, and I can watch it in my own time as many times as I feel necessary’. (Mother of a 5 year old boy)

‘I am not educated, so if you show me or explain to me how to do it, only then I can do it’. (Mother of a 5 year old girl)

## Discussion

This formative research explored carers’ perspectives about the management of intellectual and developmental disorders in the community through a network of family volunteers. The key findings of this research are that there is a huge gap between the needs of the carers and available services. Carers would welcome a volunteer-led service, and some community members would have time to volunteer. Raising community awareness in a culturally sensitive manner prior to launching such a service and linking it to the community health workers programme would increase the likelihood of success. Gender-matching was important. It would be possible to form family networks around the more motivated volunteers, with support from local non-governmental organizations. The carers were receptive to the use of technology to assist the work of the volunteers as well as for networking.

Helping others by using one’s own experiences is an established practice in the voluntary sector and is increasingly being used to support public sector services. The term peer has often been used to describe those who have similar characteristics as the target population and/or use their own experience of overcoming an illness to help others [18]. These common characteristics may include: age, gender, health concern, socioeconomic status, religion, ethnicity, locality, culture or education and these allow the peer to relate to the individuals they are offering support to [19]. Peer volunteering programmes can be informal (natural lay helpers i.e. family members/friends), or peers participating in peer-run programmes and peer employees [20,21]. Such informal support has been provided in diverse community or clinical based settings through individual sessions, group sessions or via telephone or computer [18]. Peer volunteers have also been used to deliver specific mental health interventions for various conditions such as perinatal depression, schizophrenia and bipolar disorder, substance abuse and affective disorders [22]. The results have been favourable, and lend support to the use of such approaches. However, all of these studies have been carried out in high income countries.

Peer-volunteers working alongside health services have been tried in low-income countries with physical health conditions. The most significant recent example is the use of women’s participatory groups to improve birth outcomes in Nepal [23] and India [24]. In both studies the participatory women’s group lowered the neo-mortality rates significantly in intervention groups as compared to control groups. In Bangladesh [25] and Uganda [26] peer counselors, were used effectively in delivering exclusive breast feeding programmes to mothers at home. In both studies, mothers found peer-volunteers approachable, helpful, able to relate to them and beneficial. Likewise peer counselors found the experience rewarding and felt that it has increased their status.

The use of peer (or family) volunteers for intellectual and developmental disorders is recommended by the CBR model, but presents special challenges, some of which have been explored in this study. Such conditions are often associated with poverty and stigma, and families are often isolated. However, family level cooperation and support within kinships in many rural communities like in Pakistan is generally high [27] and advantage could be taken of this cohesion to organise such groups. Furthermore, in addition to the altruistic and religious motivations, some form of material incentive, even if tokenistic, might have to be considered to make such volunteerism sustainable.

The study shows that there are social and cultural sensitivities that would need to be considered while organizing such networks, but carers also made useful suggestions about how potential barriers could be overcome. Partnership with the community health worker programme and alliances with the mosque and local faith healers could benefit the programme. This demonstrates the benefits of conducting participatory formative research prior to launching such initiatives. A spin-off from this research was that some of the participants agreed to become a part of the research team in order to assist the implementation phase the of Family Networks project.

Even in this poor part of rural Pakistan, the receptivity towards the use of technology to assist such a network was notable. It is feasible to use simple user-friendly devices to aid both training and networking. However, adapting evidence-based specialist delivered best practice in a simplified technology-supported format that can be used effectively by minimally trained family volunteers is a challenge. Furthermore, such adaptations must take into account the carers’ existing belief systems coping strategies and build on positive practices while gently challenging negative ones.

## Conclusion

In conclusion, family volunteers collaborating to deliver evidence-based packages of care after appropriate training is a feasible system that can help reduce the treatment gap for childhood intellectual and developmental disorders and can be a practical way forward in designing a CBR programme for children with intellectual disabilities and developmental disorders. However, such systems can only succeed if local sensitivities are addressed, there is willingness, and if appropriate training, resources and supervision mechanisms are put in place.

## Abbreviations

FaNs: Family networks; LHWs: Lady health workers; BHU: Basic health unit; CHWs: Community health workers; IRB: Institutional review board; CBR: Community based rehabilitation; mhGAP: Mental health gap intervention guide; LHV: Lady health visitor.

## Competing interests

The authors declare that they have no competing interests.

## Authors’ contributions

SUH and NA designed and conducted the study, and wrote the first draft of the paper under AR’s supervision, MT assisted SUH and NA in data collection and write up, FM, ZI and AR supervised the process of data collection, analysis, and report writing, AR conceived the study and finalized the manuscript. All authors read and approved the final manuscript.
